# “Our commonality is our past:” a qualitative analysis of re-entry community health workers’ meaningful experiences

**DOI:** 10.1186/s40352-015-0031-5

**Published:** 2015-12-22

**Authors:** Precious Bedell, John L. Wilson, Ann Marie White, Diane S. Morse

**Affiliations:** Department of Psychiatry, University of Rochester School of Medicine, 300 Crittenden Blvd., Rochester, NY 14642 USA

**Keywords:** Community health workers, Qualitative analysis, Re-entry services, Thematic analysis

## Abstract

**Background:**

Re-entry community health workers (CHWs) are individuals who connect diverse community residents at risk for chronic health issues such as Hepatitis C virus and cardiovascular disease with post-prison healthcare and re-entry services. While the utilization of CHWs has been documented in other marginalized populations, there is little knowledge surrounding the work of re-entry CHWs with individuals released from incarceration. Specifically, CHWs’ experiences and perceptions of the uniqueness of their efforts to link individuals to healthcare have not been documented systematically. This study explored what is meaningful to formerly incarcerated CHWs as they work with released individuals.

**Methods:**

The authors conducted a qualitative thematic analysis of twelve meaningful experiences written by re-entry CHWs employed by the Transitions Clinic Network who attended a CHW training program during a conference in San Francisco, CA. Study participants were encouraged to recount meaningful CHW experiences and motivations for working with re-entry populations in a manner consistent with journal-based qualitative analysis techniques. Narratives were coded using an iterative process and subsequently organized according to themes in ATLAS.ti. Study personnel came to consensus with coding and major themes.

**Results:**

The narratives highlighted thought processes and meaning related to re-entry CHWs’ work helping patients navigate complex social services for successful re-integration. Six major themes emerged from the analysis: advocacy and support, empathy relating to a personal history of incarceration, giving back, professional satisfaction and responsibilities, resiliency and educational advancement, and experiences of social inequities related to race. Re-entry CHWs described former incarceration, employment, and social justice as sources of meaning for assisting justice-involved individuals receive effective, efficient, and high-quality healthcare.

**Conclusions:**

Health clinics for individuals released from incarceration provide a unique setting that links high risk patients to needed care and professionalizes career opportunities for formerly incarcerated re-entry CHWs. The commonality of past correctional involvement is a strong indicator of the meaning and perceived effectiveness re-entry CHWs find in working with individuals leaving prison. Expansion of reimbursable visits with re-entry CHWs in transitions clinics designed for re-entering individuals is worthy of further consideration.

## Background

There are 2.2 million individuals currently incarcerated within jails and prisons in the United States criminal justice system; over 600,000 are released annually (Bureau of Justice Statistics 2014). Most individuals released from incarceration face difficulties upon re-entry into communities, including accessing primary care and substance abuse treatment, obtaining employment and housing, and navigating other needed social services (Kulkarni, Baldwin, Lightstone, Gelberg, and Diamant 2010; Morse et al. 2014; Wang et al. 2012). The aforementioned difficulties, if not addressed upon release, can play a role in future incarceration and recidivism. For instance, untreated health problems affect up to an estimated 80 % and 90 % of formerly incarcerated men and women, respectively, and resulting despair can contribute to drug use and the adoption of illegal subsistence survival strategies (Brewer and Heitzeg 2008). Chronic medical and mental health issues prevalent among recently released individuals include substance use disorders (SUDs), Hepatitis C, HIV, diabetes, sexually transmitted infections (STIs), cancer, suicide, and cardiovascular disease (Binswanger, Krueger, and Steiner 2009; Mallik-Kane and Visher 2005). Moreover, the risk of mortality from drug overdose and other causes in the first two weeks following release from incarceration is 129 or 12.7 times greater, respectively, than that of the general population (Binswanger et al. 2007).

Re-entry community healthcare workers (CHWs) offer strategies to address the myriad of health problems by linking recently-released individuals to healthcare and providing culturally informed assistance with systemic barriers at re-entry (Lemay et al. 2012; Love, Gardner, and Legion 1997). Hiring community members to render certain basic health services to the communities from which they come has been commonplace for at least 50 years (Lehmann and Sanders 2007). Originating within Native American communities, early formal community health worker programs in the United States were intended to improve knowledge of healthcare and behavior in tribes at risk for incarceration, illness, disability, and death (Berthold et al. 2009). Justice-involved individuals’ lives mirror those of Native Americans with respect to vulnerabilities to these risk factors (Binswanger et al. 2007). Today, CHWs often live in communities where they work, are valued and trusted members, and are in a knowledgeable position to authentically engage individuals regarding health, provide health education, and promote health (Becker et al. 2004; Maes, Closser, and Kalofonos 2014). Recent studies indicate that community health outreach is growing as a respected professional role in local communities and in the healthcare field (Berthold et al. 2009). According to the Bureau of Labor Statistics 2010 Standard Occupational Classification system (21–1094), CHWs “assist individuals and communities to adopt healthy behaviors by conducting research and advocating for individuals and community health needs” (United States Department of Labor: Bureau of Labor Statistics 2010). Community-centered outreach approaches are useful in engaging communities with histories of health disparities and offer promise for bridging underserved individuals to needed healthcare services.

Following the success of community health worker implementation in other marginalized populations, a recent initiative by the Transitions Clinic Network (TCN) beginning in 2006 employs and trains former justice-involved individuals as re-entry CHWs to provide healthcare services to individuals recently released from incarceration (Wang et al. 2010). With personal histories of incarceration, re-entry CHWs can “mitigate the effects of incarceration by fostering social support, linking formerly incarcerated individuals with existing community resources, and acting as agents for social change” (Willmott and van Olphen 2005). While there are pilot data on the health impacts of the program (Wang et al. 2010), the effects of and impacts upon the justice-involved CHWs themselves have not been elucidated in the literature. Understanding the meaning that CHWs find in their work is essential as additional former justice-involved individuals are recruited for health-related initiatives and future training programs are developed for CHWs.

Substantial research demonstrates that CHWs help to improve health care outcomes by increasing access to care and improving continuity of care (Witmer, Seifer, Finocchio, Leslie, and O'Neil 1995). Re-entry CHWs in particular reduce individuals’ reliance on costly emergency department visits for acute care, as they help patients gain knowledge regarding how to access and utilize transitions clinics providing primary care (Wang et al. 2012). Moreover, they link individuals with much-needed healthcare while considering systemic barriers that may impact individuals who were incarcerated. Elucidating the aspects of re-entry CHWs’ roles that are meaningful to them is an important early step in demonstrating potential benefits that will justify efficacy, effectiveness, and implementation research in this specific group of patients and CHWs.

While the literature has examined the role and effectiveness of CHWs in other populations, there is a significant gap regarding the nature of the re-entry CHW experience and how it may contribute to improved health outcomes and continued cost savings for healthcare systems. More specifically, the perspectives of re-entry CHWs on their roles are currently unexplored in the literature. Therefore, this study investigates these questions through a qualitative analysis of the lived experiences of re-entry CHWs in the TCN.

## Methods

### Study sample

Fifteen re-entry CHWs employed by the TCN, from varied geographic regions, and active in TCN’s national training events were invited to participate in the study. The TCN is a national consortium of 11 clinics in 10 states providing healthcare to individuals upon leaving incarceration. The consortium also facilitates a Post-Prison Healthcare Worker Certification program administered by the City College of San Francisco to train and professionalize CHWs. The certification training provides CHWs with knowledge and skills regarding healthcare delivery and healthcare management for individuals released from incarceration. The CHWs’ work includes extensive outreach prior to and following release from incarceration.

Of the 15 re-entry CHWs, 12 agreed to participate in the current study. The number of re-entry CHWs in the U.S. has not been reported in the literature; however, the majority (80 %) of CHWs employed by the TCN enrolled in the present study. Demographic data for the 12 CHWs are provided in Table 1. Among the 12 participating re-entry CHWs, seven were women and five were men; nine identified as African-American, two as Caucasian, and one as Hispanic. Ages ranged from 33 to 74. Most CHWs have had some college education; many earned academic degrees while incarcerated.

**Table 1:** Demographic data of study participants (*n* = 12)

Pseudonym	Gender	Age	Race/Ethnicity	Primary Affiliation	Years Since Incarceration	Geographical Region (U.S.)	Education
Alexander	Male	40–49	Hispanic	Hospital	≤5	Northeast	B; CHW certification
Paul	Male	40–49	African-American	Clinic	≥15	West	C; CHW certification
Maya	Female	40–49	African-American	Community agency	6–10	Northeast	B; CHW certification
Jasmine	Female	30–39	Caucasian	Clinic	≥15	West	A; CHW certification
Sarah	Female	50–59	African-American	University	6 – 10	Southeast	D; CHW certification
Abdullah	Male	40–49	African-American	University	Not available	Mid-Atlantic	C
Johanna	Female	50–59	African-American	Community agency	≤5	Northeast	D; CHW certification
Elijah	Male	50–59	African American	Community	6 – 10	West	B; CHW certification
Michael	Male	30–39	African-American	University	Not available	Northeast	CHW certification
Mona	Female	50–59	African-American	Community agency	6 – 10	West	A; CHW certification
Florence	Female	70–79	Caucasian	University	≥15	Northeast	A
Michelle	Female	60–69	African-American	University	≥15	Northeast	D; CHW certification

A) High school or equivalent (e.g. GED)

B) Some undergraduate training

C) Bachelor’s degree

D) Graduate school degree

### Data collection

The authors utilized two main approaches for collecting narratives among the 12 CHWs who agreed to participate in the study. First, study participants were encouraged to recount salient experiences in a journal-like manner to facilitate recall of specific encounters and emotions during their tenure as CHWs. This style of reflective journal writing is a well-cited qualitative research technique and enables researchers to “clarify, reinterpret, and define” the experiences described in the written text (Andrusyszyn and Davie 2007; Janesick 1999). A brief workshop was scheduled at the end of a national conference, during which five of the 12 CHWs wrote narratives. As an alternative to this face-to-face method, two later sent narratives via email and five CHWs used a subsequent online forum to submit responses.

As a CHW of the TCN network led data collection efforts, respondents’ narratives emerged in a context of ongoing interactions and within a variety of naturalistic and ecologically valid settings during the study period. Interactions were both structured (e.g. professional conferences, online training forums, emails, and phone conversations) and unstructured (personal communication). The additional interactions contextualized the data collection naturalistically and provided the research team with opportunities to: a) review respondents’ submissions for clarity and comprehension; b) seek deeper understanding about their written experiences by discussing submissions as these relate to the rich context of CHWs’ lives; and c) conduct respondent verification around key emerging themes. CHWs’ responses averaged two pages in length and were submitted in various formats, including poetry and essays. Additionally, the CHW participants reported demographic information. The University of Rochester Research Subjects Review Board deemed the study as low risk and did not require written consent.

### Analytic approach

A multidisciplinary analytic team identified recurrent events and terms within the narratives; the team included a formerly incarcerated CHW employed as a re-entry specialist and researcher, an internal medicine physician and researcher with expertise in treating populations served by re-entry CHWs, a community-engaged researcher in a Department of Psychiatry, and a research associate at the bachelor’s degree level with extensive training in qualitative analysis and experience with justice-involved populations. The team conducted a qualitative thematic analysis utilizing an iterative coding process (MacQueen, McLellan, Kay, and Milstein 1998) and organized the data using ATLAS.ti (Smit 2002). Quotes from participants’ narratives were assigned one or more codes based on content relating to their experiences as CHWs until thematic saturation was achieved. Recurring codes were then organized into higher order conceptual themes (Creswell 2012). One of the co-authors conducted the first round of analysis and coding, and the remaining co-authors reached consensus with the primary coder. The co-authors agreed upon final coded quotes, codes, and themes. All participants were assigned pseudonyms. Numbers of quotes coded under each of the themes and corresponding percentages are presented in relation to all coded statements in Table 2.

**Table 2:** Numbers and percentages of quotes from meaningful experiences narratives relating to the 6 conceptual themes

Quote distribution according to theme and subtheme	*n*	%
Advocacy and support	36	9.94
Desire for advocacy	16	4.42
Working to affect social change	20	5.52
Empathy relating to CHWs’ history of incarceration	66	18.23
Building trust	5	1.38
CHWs’ experience while incarcerated	16	4.42
CHWs’ former incarceration and arrests	14	3.87
Empathy	12	3.31
Past hardship	19	5.25
Giving back	12	3.31
Giving back	12	3.31
Professional satisfaction and responsibilities	117	32.32
Difficulties and uncertainties surrounding CHW role	4	1.10
Meaningful experiences as CHWs	19	5.25
Professionalism	24	6.63
Resource navigation	9	2.49
Role of CHW	33	9.12
Satisfaction from CHW position	24	6.63
Therapeutic alliance	4	1.10
Resiliency and educational advancement	79	21.82
CHW certification	10	2.76
Educational advancement	8	2.21
Motivation and goal-seeking	21	5.80
Personal strength	17	4.70
Personal success	23	6.35
Social and racial injustices	52	14.36
Problems with jails, prison, and the justice system	17	4.70
Social inequities	35	9.67

## Results

Six major themes emerged from the narrative data following coding: 1) advocacy and support; 2) empathy emanating from a personal history of incarceration; 3) giving back to recently released individuals; 4) professional satisfaction and responsibilities; 5) personal resiliency and educational advancement; and 6) experience of social and racial injustices. Quotes pertaining to these six themes are provided in Table 3 in addition to those described below.

**Table 3:** Examples of quotes pertaining to the 6 conceptual themes

Advocacy and Support	“I use this pain to fuel my knowledge base and to become more committed to the cause of creating a human rights movement that Dr. Michelle Alexander envisions. It’s really not about ending prisons for public safety or to reduce costs, it’s about the deliverance of a nation. I have spoken on these themes throughout this certification.” *Michelle*
Empathy Relating to CHWs’ History of Incarceration	“As I view our stories I reflect on our circle at the time. Most of us did not know what to expect, but once we started sharing our experiences we understood how much the experience would impact our lives forever. It was an emotional healing experience. It was raw and uncut also.” *Mona*
Giving Back	“To become a true advocate, I must feel the pain, the frustration of the inequities still faced by so many of us, including women, to stay the course. Internal reflection brings on, for me, a true meaning of doing that right thing, for the right reason. When I speak or have spoken on my commitment to the movement, how I am and want to continue to be a part of what Dr. King started.” *Michelle*
Professional Satisfaction and Responsibilities	“I know I need to find a balance for myself in this area, and practicing the skills that I have learned this week, and that I am learning from this course, will assist me in my goal of becoming a better CHW and a better person overall.” *Michael*
Resiliency and Educational Advancement	“What it means to be a true re-entry CHW is to meet people where they are at, in their time of need, when they need us the most. And to help navigate, motivate, and advocate…” *Sarah*
Social and Racial Injustices	“I think it persists because of the mentality of those that are in a position to change the policies. The mentality would need to change from furthering the punishment of being incarcerated, to that of how [to] bring the rate of incarceration down.” *Alexander*

### Advocacy and support

Community health workers discussed their advocacy and support on behalf of patients and how it is a form of activism. Their advocacy and support are uniquely informed by personal knowledge and experiences. Narratives indicated that CHWs recognized that patients needed advocates to help them access medical and community resources. Patients also needed support because they either did not know how to access resources or prior discrimination made them mistrustful of formal health and human services within communities. Two CHWs described personal experiences that created a trusting relationship and advocated for positive change:

*“Most people from urban communities have never had anyone help them without anything in return…therefore, there is always distrust and when they know that they have someone guiding them who has their best interest in mind and does not ask for anything in return, they are able to learn a new reality.”*

*Alexander*

Alexander revealed that advocacy and support were his primary motivation in becoming a CHW, since many people had not helped him in the past, and he himself lived in poor urban communities most of his life. Distrust of healthcare systems is embedded within urban cohorts; having been in similar situations, he connects with patients easily.

*“It affected me personally, because I needed an advocate, someone to speak up for me, to make a difference and bring about a positive change. Today, I am a Community Health Advocate. I am in a position to make a positive change in my patients’ lives. I am that voice for them and encourage them to use their voices to speak up for themselves.”*

*Maya*

Maya used herself as an advocate and role model to support patients in making choices that will help them move forward by speaking out against discrimination and injustice. Another CHW spoke about her experience of supporting patients by identification:

*“I am able to engage a potential new patient from the very beginning because of our shared walk, past, and/or experience.”*

*Joanna*

Joanna continued transferring her skills in a positive way as a committed advocate for her patients. Providing both advocacy and support in the setting of scarce substance abuse treatment resources was an ongoing process for another CHW:

*“I kept calling her…and going over to her house…but to no avail…Finally, I caught up with this woman and she had a change of heart. She wanted to go [to] inpatient [addiction treatment] and I was almost afraid to act on that request because her track record was so bad but I…got her on the waiting list. She called me faithfully every other day to find out what her status was and both my fingers and toes were crossed hoping that this was it. Her day finally came and she was admitted to the rehab; everyone on our team was overjoyed. I called periodically to see if she was still there and she was, and I might add she was doing well.”*

*Florence*

Florence described herself as the caretaker for women who have suffered severe trauma as she has. As noted above, she worked to be ever-present in caring and providing resources for her patients. The CHW below described his experience while advocating for a person who had served six years in solitary confinement and has mental health issues.

*“My first job was to get him housing…His communication and social skills were none…[the programs rejected him]. I did an application for him for disabled housing which usually takes 6–9 months, sometimes a couple of years. But I developed a rapport with the staff and told them his story and that we have been trying to get him housing. They appreciated all of my dedication and hard work I did for him and got him into a brand new building with direct services downstairs.”*

*Paul*

Paul described advocating for many years to help others find housing. He reported working well with patients, which he attributed to his personal knowledge of homelessness and incarceration.

### Empathy relating to CHW history of incarceration

CHWs gave examples of connecting to service providers upon release and knowing how challenging that was from personal experience. One of the CHWs illustrated below:

*“I come from the same background as my clients. So I relate [be]cause I have been there. The tone of my voice is soft and my attention is completely on them, and they can feel my genuine heart, caring, and compassion, improv[ing] my work.”*

*Paul*

CHWs indicated that they empathize with patients’ struggles, the pathways that lead to prison, and the trauma and stigma of being labeled a “felon.” CHWs also addressed social determinants of health and health disparities using empathy.

*“…And most of all, to inspire our people that come from the same place we once were to make a difference in their community in which they live. And to help teach them to make healthy choices and take care of their whole well-being in spirit, in flesh, [and] mind. Re-entry CHW is not only a profession, but is a true passion straight from the heart that moves many lives to make change.”*

*Jasmine*

Another CHW addressed stigma with understanding:

*“I can utilize my experiences to advocate and promote healing to a population of people that are forgotten and deemed as lepers, not to be touched and left to exist in a world that does not want them.”*

*Sarah*

Sarah described how she interacts and connects with patients, particularly women with similar trajectories.

Another CHW applied logic and empathy to draw upon her own self as an example to promote resiliency post-incarceration:

*“My logic was, who better than myself, someone who has walked the walk and now stand[s] as a power of example that incarceration does not define who I am.”*

*Joanna*

One CHW described being both a role model and a source of hope for those with whom he empathized:

*“Because I love it. I just like to help people. I see people and [say], ‘I was a whole lot worse. So if I can change, it's in you. But you have to dig deep.’ See, a lot of times what happens is people give up hope. So if you have someone who's trying to give you some hope, then maybe you can do it.”*

*Paul*

### Giving back

CHWs viewed giving back as influential in wanting to pass on the health information and health literacy that they and their community members did not previously receive. They touted the importance of ending health disparities:

*“Some future plans that I have [are] to bring more health education to the inner city youth. There is not enough real life health awareness given to the youth…it is instrumental they are given the right guidance prior to adulthood. Speaking on personal experience, I didn’t understand the importance of keeping up with my health and so didn’t the people around me.”*

*Alexander*

The CHWs described how their own painful experiences as formerly incarcerated people guided and motivated the hard work needed to improve the health of the re-entry population.

*“It takes work to remove those barriers and build trust, then to remove the barriers enough for the doctors to work comfortably with the patient. [This] could only be done by a person with experience and who has lived the same lifestyle and knows some of the pain, neglect, and prison life.”*

*Sarah*

### Professional satisfaction and responsibilities

CHWs reflected on experiences of professional satisfaction while serving in their role:

*“Can you think of anything that is more joyful or rewarding than to see a fellow human being rise up from the dust and the dirt of addiction and what goes with it to become who they were meant to be?”*

*Florence*

CHWs noted that their professional role and training complemented those of other medical providers to close the gap of discrimination and bias that patients experience.

*“As a CHW, I am out in the ‘trenches’ with our patients. I am with them and see how they interact with those in the community. I am with them when they go apply for public assistance and social security benefits, and I am witness to how they are treated by those who are not in favor of giving second chances.”*

*Joanna*

*“We are the [part of the] equation that dampens recidivism and puts health priorities back into the lives of returning citizens and their families. We are the educators that combat myths and ignorant beliefs that people live with for many years. We are the true driving force behind re-entry health and education.”*

*Alexander*

One CHW described an ongoing project collaborating with businesses, community leaders, and medical professionals to house and provide services to formally incarcerated people with chronic conditions, incorporating the client-centered principles learned in the certification program. Another participant perceived that her patients’ needs are primary:

*“I have to get the goals of my clients fulfilled and will align all my resources so that the client [can] move forward and complete the task.”*

*Mona*

Mona’s described her strategies in group facilitation and motivation to develop relationships with her patients, consider individuals’ circumstances, and empower them.

### Resiliency and educational advancement

Mona described urgent needs for education and change in the black community in the context of adversity, racial injustice, and the need to believe in oneself:

*“I live in a community where so many black men hate themselves and consider themselves [so] insignificant that it is easy to kill, [and] racial profiling is evident and prevalent. Going to prison is not a trend- it is a reality for the black male, and the sooner we invest in ourselves, communities and each other, by educating ourselves to knowing thyself to be true is when change will come. We must go back and fix what is broken and start believing in ourselves again or we will continue to be enslaved as a people.”*

*Mona*

She described a resiliency grounded in a belief in herself and that change is possible. One CHW wrote an inspiring poem alluding to his role in his family and community, staying positive and healthy, and being a CHW, which are his protective factors.

*“Dream healthy things for our friends and family.*

*Just think outside the box.*

*Give [us] a push to grow to be [a] better leader, father, husband, friend, brother, sister, mother. CHW: a better ME.”*

*Abdullah*

Abdullah used poetic devices to express what is meaningful for him as a CHW and an individual. The CHW below noted the importance of educational advancement in the post-prison CHW training program given the context of mass incarceration and resulting impacts on a community of people.

*“I plan to complete the [CHW training] course by pushing myself, remembering the purpose and…how this will add to my resume and portfolio. This course is meaningful for me because it informs me on the issues of mass incarceration, how they affect the healthcare of formerly incarcerated people, and how I can reach them to help provide assistance.”*

*Michael*

Michael noted his concern for mass incarceration as a major impetus in pursuing employment as a CHW. Another CHW credited his resiliency to his own lived experiences:

*“Let me say the journey to get here has been tiring, trying, blaming, living in denial for some many years and yet so wonderful that I would not change a thing. It has taught me the meaning of gratitude.”*

*Elijah*

Elijah’s candid remarks highlight the roles of resiliency and learning from mistakes for re-entry CHWs.

### Social and racial injustice

Issues related to race resonated throughout the narratives and themes, especially in reference to the social determinants of health, health disparities, and how race is a factor in mass incarceration. One CHW noted:

*“In looking at the analysis of [people incarcerated in] prisons and jail…, the numbers are extraordinarily different between the groups. Not much has changed for this population of people since slavery in the early 1800s… I would consider it to be worse than slavery, especially with all the education, opportunity and advances we have made as humans in America. We stand on the American Dream and Land of the Free, but for who?”*

*Mona*

One CHW wrote about the impact of racism upon a systemic and personal level and of her unwavering commitment to change:

*“I cited Angela Davis and her mission and devotion to ending incarceration and how that would be a small compensation for slavery and all its vile evils. To have been part of a system that sentenced me so differently than a white woman and now being able to help others, as I did on the inside, is testimony to how I did not allow those years of bondage to break my spirit.”*

*Michelle*

The injustice of incarceration and the criminal justice system resonated with CHWs’ goals and accomplishments. Being poor and/or a person of color and returning to communities that lack resources to support successful re-entry inspired the CHWs to advocate for better treatment in healthcare systems. CHWs upon patient health outcome, not on patient outcomes:

*“Being a participant/observer researcher, policy and reform lobbyer, and an advocate who is part of the national movement that is fighting to change the criminal justice system and its treatment of those it incarcerates, it angers me and it saddens me to understand the subliminal, yet obvious, tactics the criminal justice [system] has taken to annihilate a race of people. There is no other way to interpret what is taking place within this country when it comes to the rate of incarceration of poor, disenfranchised, marginalized people, who happen to be of color.”*

*Alexander*

Michelle identified with varied change agents for social inequities:

*“I am part of many communities, people of color, people of continuing education, and a community of writers, part of a community of healthcare workers and public health workers, part of several movements that advocate for equality. This is very meaningful to me for many reasons; the important one is that we are all fighting for the human rights of people without voices.”*

*Michelle*

## Discussion

### Major themes

This study explored what is meaningful for former justice-involved CHWs who assist patients recently released from prison with needed services and support. These CHWs routinely visit prisons and jails to recruit and inform potential patients about healthcare services available to them. After serving time, some as many as 27 years, why would formerly incarcerated individuals want to keep alive a connection to the criminal justice experience? The authors investigated this seeming paradox by analyzing 12 meaningful experience narratives generated by re-entry CHWs.

The re-entry CHWs reported advocacy and support as meaningful components of their professional position. They described providing additional support to individuals recently released from incarceration regarding social and behavioral issues that impact upon health, such as housing and addiction treatment. The re-entry CHWs wanted to help patients overcome the barriers associated with re-entry and were dedicated to advocating for patients, providing unconditional support, and walking the journey of sobriety (when needed) toward the mutual goal of successful reintegration. Moreover, they aimed to establish rapport with patients and link them with long-term physical and mental healthcare through various Transitions Clinics. Once longitudinal patient data on the TCN are collected, the impact of re-entry CHWs on patient health outcomes will be better understood.

Empathy emanating from a personal history of incarceration also resonated throughout the narratives. Unlike most professions, a personal history of incarceration and re-entry is a requirement for the CHW position. The TCN not start with the acronym TCN. Rather than being a barrier, incarceration history is a necessary job qualification and asset in the TCN, thus recasting the typical stigma, shame, and inability to meet daily needs associated with having a history of incarceration (Harris 2006). Empathy is also considered to be a cornerstone of cultural humility and patient-centered care (Tervalon and Murray-Garcia 1998). The narratives are poignant examples of how medical systems can provide culturally-specific care to help end health disparities (Maes, Closser, and Kalofonos 2014).

Re-entry CHWs recounted empathy for patients as they reflected upon unresolved deep frustrations from their own personal experiences following release from prison and their own retraumatization. Study participants may continue to cope with struggles related to prior experiences, including those which precipitated their initial incarceration. Substance abuse problems have been shown to lead to recidivism (Staton, Leukefeld, and Webster 2003). Additionally, mental health symptomology as a result of childhood abuse, intimate partner violence, sexual assaults from police or prison guards, and lack of housing and employment can mediate recidivism and threaten health (Cottler, O’Leary, Nickel, Reingle, and Isom 2014; Reingle-Gonzales and Connell 2014; Makarios et al. 2010; Staton, Leukefeld, and Webster 2003). Among reentry CHWs to maximize an understanding of how to best support CHWs in their work. Future investigation could more explicitly examine trauma histories among re-entry CHWs to maximize understanding how best to support CHWs in their work.

Giving back to patients was described as an important component of the CHW experience. Study participants acknowledged the lack of linkages and bridges that, if present, could have benefited the CHWs’ successful reintegration. Only a few descriptive studies have explored how to eliminate systemic barriers to “bridging” medical healthcare providers with substance abuse treatment staff in an effort to improve patient health outcomes (Wenzel, Longshore, Turner, and Ridgely 2001; Wenzel, Turner, and Ridgely 2004), a model that continues to lag in implementation (Morse et al. 2014). However, re-entry CHWs expressed gratification in connecting patients with TCN healthcare providers. CHWs forge a similar “bridge” to long-term primary care, inform potential patients of healthcare-related resources upon release, and provide timely social support as individuals navigate the murky fields of re-entry that may put them back in proximity to the community environmental factors (described in the study narratives) that can precipitate recidivism (Maes, Closser, and Kalofonos 2014). Giving back what they received or should have received while re-entering communities is profoundly meaningful to the re-entry CHWs. Although the CHWs want to give back, it is important to note that many individuals’ parole requirements include not being allowed to associate with other formerly incarcerated people (NYS Department of Corrections and Community Supervision 2015) (https://www.parole.ny.gov). The peer support which CHWs provided and described may be withheld from some people due to this requirement and is worthy of further examination as a potential systemic barrier to health improvement.

Study participants cited professional satisfaction and responsibilities as salient experiences while employed as re-entry CHWs in the TCN, advancing beyond expectations. Development of professional satisfaction in meeting job expectations is a key issue for those who may be in their first professional-level job due to the “school to prison pipeline” that negatively impacts students in schools from communities with high poverty and people of color (Wald and Losen 2003). Employment is known to help prevent recidivism, so the model of formerly incarcerated CHWs can be mutually beneficial (Davis 2013). As professionals, CHWs also serve as role models and mentors offering promise to other individuals with criminal histories. Re-entry CHWs described building collaborative relationships with community stakeholders in an effort to enhance re-entry services and establish themselves as community leaders. Community relationship building simultaneously helped the re-entry CHWs feel successful and capable of becoming agents of social change. One such re-entry CHW (and study P.I.) is the founder and director of a center that provides advocacy, support, information, and resources to families who have relatives or close friends in prison (Bedell 2014). Re-entry CHWs are thus in a unique position to advocate for policies that benefit communities as they continue their own professional development.

Re-entry CHWs emphasized the importance of personal resiliency and educational advancement during their tenure. Personal advancement and education usually precede professional development, but become a post-incarceration task for those who miss the opportunity to obtain an adequate education. Re-entry CHWs recalled being persistent and resilient in the face of thwarted education and vocational opportunities. Resilience has been associated with mentoring (Zimmerman, Bingenheimer, and Notaro 2002), and in this case, the re-entry CHWs receive mentoring practice during trainings to benefit clients. The narratives spoke to the dedication that re-entry CHWs have to improve their lives as professionals, and in turn, the lives of others. They continued to pursue higher education and gain a critical consciousness about systemic oppression that can inspire action and respect in their communities (Freire 1993). It is important to note that throughout the data collection period, the TCN re-entry CHWs were enrolled in the Post-Prison Health Certification program through the City College of San Francisco (2014) (http://www.ccssf.edu). The re-entry CHWs reported the significance of increased knowledge for themselves as they continue their professional development.

Re-entry CHWs’ experiences and understanding of social and racial injustices were poignantly stated throughout the narratives. The underpinnings and aftermath of racism and discrimination (not of race) are well-documented as core causes of incarceration (Alexander 2010). Internalization of poverty and disenfranchisement may impede individuals’ abilities to negotiate valuable resources needed for successful re-entry (Morse et al. 2014). The finding that racial inequities were an overarching theme throughout many of the narratives was not surprising, as 77 % of the current study’s re-entry CHWs are people of color, who are disproportionately incarcerated (Clemons 2014): one in 15 black men are currently incarcerated in the U.S., compared to one in 36 Hispanic men, and one in 106 white men (Barnert, Perry, and Wells 2014). Although the Post-Prison Health certification course raised the issue on an academic level, it is evident that all shared a personal and passionate connection. The re-entry CHWs emphasized dedication to fight for the key issue of equality and justice within their professional roles. A recent qualitative study of CHW narratives identified social justice as the driving force in what was meaningful to CHWs (Murray and Zeigler 2015).

The Transition Clinic Network consortium is a project funded by the Center for Medicare and Medicaid Services (CMS) under the Affordable Care Act. At a time when CHWs are one of the fastest growing professions (US Bureau of Labor Statistics, Occupational Outlook Handbook 2009), there is currently no service-level reimbursement for re-entry CHWs to see patients. As CMS administrators consider funding such reimbursements or including CHWs in its portfolio of population-level healthcare approaches, it will be important to consider the potential health impacts (Barnert, Perry, and Wells 2014; Patel et al. 2014). Furthermore, cost-benefit analyses could be conducted to examine potential savings associated with implementing similar CHW-based programs that link recently released individuals to long-term primary care.

There are growing bodies of research that acknowledge the panoply of key functions CHWs perform (Witmer, Seifer, Finocchio, Leslie, and O'Neil 1995). Despite the potential significant impact that CHWs have had upon on improving healthcare quality and thus outcomes for vulnerable and marginalized populations, mainstream funding continues to be the barrier that thwarts widespread hiring of CHWs, particularly, the re-entry CHW. Moreover, funding sources for CHWs are mainly time-limited grants. This study provides important information to support advocacy for funding opportunities for Transitions Clinics and community stakeholders. Additionally, hiring, professionalizing, and paying CHWs a fair wage is a systems-level intervention to address health disparities especially among communities whose healthy social ties have been decimated by inordinate rates of resident incarceration and years of racial inequality (National Health Care for the Homeless Council, 2011).

### Limitations and strengths

CHWs sampled live and work in several different regions across the U.S. They recounted experiences with multiple cohorts in communities with varied community health and support agencies. While the current study was successful in exploring meaningful experiences among a diverse group of CHWs, there are some important limitations. The narratives were written, rather than gathered via face-to-face semi-structured interviews, an alternative approach that might have yielded more detailed data. Also, the study examines the viewpoints of the CHW, but does not include the view of the patients they serve regarding the CHWs. Despite the above limitations, this study provides an important early step in garnering systemic information to advance the implementation of CHW-based initiatives that address the health needs of recently released individuals.

## Conclusions

Re-entry CHWs find meaning in their work, derive professional benefits, and bring unique abilities to the tasks. They regard empathy, resilience, giving back, advocacy, and the pursuit of social justice as most meaningful in their work. Any of these qualities can bolster patient-centered care for recently released individuals, and in turn, can provide benefits to patients that improve public health in ways previously unexplored. Additional research is needed to further elucidate the perspectives of CHWs and their unique roles in eliminating health disparities associated with individuals recently released from incarceration. Furthermore, research should be directed to study cost savings that could result from utilizing re-entry CHWs, particularly with regards to healthcare and recidivism.

## Abbreviations

CHW: Community health worker
TCN: Transitions Clinic Network
